# Analyzing the Effects of Topological Defect (TD) on the Energy Spectra and Thermal Properties of LiH, TiC and I_2_ Diatomic Molecules

**DOI:** 10.3390/e23081060

**Published:** 2021-08-17

**Authors:** Peter Nwabuzor, Collins Edet, Akpan Ndem Ikot, Uduakobong Okorie, Morris Ramantswana, Ridha Horchani, Abdel-Haleem Abdel-Aty, Gaotsiwe Rampho

**Affiliations:** 1 Physics Production Technology, School of Science Laboratory Technology, University of Port Harcourt, Choba PMB 5323, Rivers State, Nigeria; peter.nwabuzor@uniport.edu.ng; 2 Theoretical Physics Group, Department of Physics, University of Port Harcourt, Choba PMB 5323, Rivers State, Nigeria; akpan.ikot@uniport.edu.ng (A.N.I.); uduakobongokorie@aksu.edu.ng (U.O.); 3 Department of Physics, Akwa Ibom State University, Ikot Akpaden, Uyo PMB 1167, Akwa Ibom, Nigeria; 4 Department of Physics, University of South Africa, Johannesburg 1710, Florida, South Africa; ramanm@unisa.ac.za (M.R.); ramphjg@unisa.ac.za (G.R.); 5 Department of Physics, College of Science, Sultan Qaboos University, Al-Khod 123, Muscat P.O. Box 36, Oman; horchani@squ.edu.om; 6 Department of Physics, College of Sciences, University of Bisha, P.O. Box 344, Bisha 61922, Saudi Arabia; amabdelaty@ub.edu.sa; 7 Physics Department, Faculty of Science, Al-Azhar University, Assiut 71524, Egypt

**Keywords:** Nikiforov–Uvarov (NU), topological defect, diatomic molecule, thermal properties

## Abstract

In this study, the impacts of TD on the energy spectra and thermal properties of **LiH**, **TiC** and **I_2_** diatomic molecules is considered. The Schrodinger equation in cosmic string spacetime is solved with the generalized Morse potential using the well-known (NU) method. The energy spectra and eigenfunction are obtained respectively. The energy spectra is used to obtain the partition function which is then used to evaluate the thermal properties of the system is evaluated accordingly. We find that the energy spectra in the presence of the TD differ from their flat Minkowski spacetime analogue. The effects of the deformation parameter and TD on the thermal properties of the system is also analysed in detail. We observe that the specific heat capacity of the system tends to exhibit quasi-saturation as the deformation parameter and topological defect approaches unity. The results of our study can be applied in the astrophysical situation where these modifications exist in the understanding of spectroscopical data and it may be used as a probe of the presence of a cosmic string or a global monopole in the Universe.

## 1. Introduction

The deficiency of the harmonic oscillator in the description of interatomic interactions in diatomic molecules brought about the Morse potential in 1929 [1,2]. The triumph of this model cannot be overemphasized, although there have been several modifications due to its shortfalls in modern spectroscopic studies. This has led researchers to propose more interaction potentials, such as Improved Manning Rosen potential [3], Frost–Musulin potential [3], Rosen–Morse potential [3,4], etc. Moreover, it is the pursuit of every molecular physicist to continuously seek a better molecular or interaction potential that perfectly simulates, as it were, the interatomic interactions in diatomic molecules. With this in mind, we are inspired to adopt an interaction potential named the generalized Morse potential (GMP), ref. [5] given as:

(1) $$V\left(r\right)=D_{0}{\left(1-e^{-\delta \left(r-r_{e}\right)}\right)}^{2}+D_{1}{\left(q-e^{-\delta \left(r-r_{e}\right)}\right)}^{2}$$

In respect to what has been observed in previous studies of molecular potential, Ikot et al. [5] modified the Morse potential to a general form so as to allow for more physical applications and a comparative analysis to existing studies with other molecular potentials. In addition, in molecular physics, researchers have, in recent times, paid great attention to obtaining a modified version of potential function by employing potential energy functions with more parameters. The results from these investigations have been found over the years to closely agree with experimental data from those with fewer parameters. This model (GMP) will be an important tool for spectroscopists to represent experimental data, verify measurements, and make predictions [6,7,8,9,10]. In recent times, researchers have paid great attention to solving the SE with various potential models, because it contains all the necessary information about the system. A number of researchers have carried out research in this direction [11,12,13,14,15,16].

Furthermore, for many years, the study of quantum dynamics of a single particle interacting in a given potential with a topological defect has been a subject of great interest to researchers. The formation of a topological defect is thought to have occurred during a phase transition in the early universe [17,18,19,20]. The effects of topological defects on the dynamics of both non-relativistic and relativistic quantum mechanical systems have recently piqued researchers’ interest, such as screw dislocation [21], bound eigenstates of electron and holes to a declination. Furtado et al. [22] studied the landau levels in the presence of a topological effect [23], a coulomb and quantum oscillator problem in conical space [24] and the hydrogen atom in curve–space time [24], etc. 

Topological defects play an important role in modifying the physical properties of many quantum systems, and they have long been a popular topic in fields such as condensed matter and gravitational physics. In gravitation, topological defects appear as monopoles, strings, and walls [25,26]. In condensed matter physics, they are vortices in superconductors or superfluids [27,28], domain walls in magnetic materials [29], solitons in quasi-one-dimensional polymers [29,30] and dislocations or disclinations in disordered solids or liquid crystals [31]. A change in the topology of a medium caused by a linear defect in an elastic medium, such as a disclination, dislocation, or dispiration, or a cosmic defect in spacetime, has a certain impact on the medium’s physical properties [32]. However, in view of these studies, no research article has yet studied the energy spectra and thermal properties of GMP in a medium with a global monopole (GM). 

A global monopole is an exotic object that may have been formed during the phase transition in the very early universe. When the corresponding vacuum is non-contractible, $M≅S^{2}$ [26]. Such an object can exist, for example, due to the spontaneous breaking of global SO(3) symmetry. Its gravitational field has been studied by Barriola and Vilenkin (BV), who found that the metric is Minkowski-like, and though not flat, it suffers from a deficit in solid angle [26,27]. 

Lately, another area of research that has gained unparalleled attention is the study of thermal properties of quantum systems [33,34,35]. It is thus very expedient to research the effect of declination on particle dynamics in a quantum system based on the information gathered [22,36,37,38,39,40,41,42,43,44,45]. However, to the best of our knowledge, no study in the literature has scrutinized the impact of the cosmic string parameter on the energy spectra and thermal properties of **LiH**, **TiC** and **I_2_** diatomic molecules. These molecules were considered for diatomic molecules because of their wide industrial applications. 

The major goal of this paper is two-fold: first, we studied the energy shift related with a non-relativistic quantum particle interacting with the GMP in the spacetimes generated by a cosmic string for selected diatomic molecules. Further, we analyzed the effects of the topological defect on the thermal these diatomic molecules.

This paper is organized as follows. In Section 2, we present a review of the Nikiforov–Uvarov (NU) method. In Section 3, we present the theory and calculations. In Section 4, we evaluate the thermal properties of the generalized Morse potential with a topological defect. In Section 4, we discuss the effects of the topological defect on the energy spectra and thermal properties of **LiH**, **TiC** and **I_2_** diatomic molecules placed in the gravitational field of a cosmic string. Finally, in Section 5, we draw some conclusions.

## 2. Nikiforov–Uvarov (NU) Method

The NU approach reduces a second-order linear differential equation to a generalized hypergeometric form equation [46,47,48,49,50,51,52,53]. The method produces a solution in terms of special orthogonal functions, as well as the energy eigenvalue. With the right coordinate transformation, s=s(r), the equation is transformed as follows [52]:

(2) $$\psi^{\prime \prime }\left(s\right)+\frac{\tilde{\tau }\left(s\right)}{\sigma \left(s\right)}\psi^{\prime }\left(s\right)+\frac{\tilde{\sigma }\left(s\right)}{\sigma^{2}\left(s\right)}\psi \left(s\right)=0$$

In order to find the solution to Equation (2), a wave function of the form [52]:

(3) ψ(s)=ϕ(s)y(s)

is used. On substitution of Equation (3) into Equation (2), the hyper-geometric equation [52] is obtained as follows:

(4) $$\sigma \left(s\right)y^{\prime \prime }\left(s\right)+\tau \left(s\right)y^{\prime }\left(s\right)+\lambda y\left(s\right)=0$$

The wave function is given as:

(5) $$\frac{\phi^{\prime }\left(s\right)}{\phi \left(s\right)}=\frac{\pi \left(s\right)}{\sigma \left(s\right)}.$$

The hyper-geometric-type function y(s) is expressed in terms of the Rodrigues relation as [52]

(6) $$y_{{}_{n}}\left(s\right)=\frac{B_{n}\left(s\right)}{\rho \left(s\right)}\frac{d^{n}}{ds^{n}}\left[\sigma^{n}\left(s\right)\rho \left(s\right)\right]$$

where $B_{n\ell }$ is the normalization constant and ρ(s) is the weight function, which satisfies the condition below:

(7) $$\frac{d}{ds}\left(\sigma \left(s\right)\rho \left(s\right)\right)=\tau \left(s\right)\rho \left(s\right)$$

where also,

(8) $$\tau \left(s\right)=\tilde{\tau }\left(s\right)+2\pi \left(s\right)$$

For bound solutions, it is required that

(9) $$\frac{d\tau \left(s\right)}{ds}<0$$

Therefore, the function π(s) and the parameter λ required for the NU method are defined as

(10) $$\pi \left(s\right)=\frac{\sigma^{\prime }\left(s\right)-\tilde{\tau }\left(s\right)}{2}\pm \sqrt{{\left(\frac{\sigma^{\prime }\left(s\right)-\tilde{\tau }\left(s\right)}{2}\right)}^{2}-\tilde{\sigma }\left(s\right)+k\sigma \left(s\right)}$$

(11) $$\lambda =k+\pi^{\prime }\left(s\right)$$

The values of k are obtained if the discriminant in the square root of Equation (10) vanish, so the new eigen equation becomes

(12) $$\lambda_{n}=-\frac{nd\tau \left(s\right)}{ds}-\frac{n\left(n-1\right)}{2}\frac{d^{2}\sigma \left(s\right)}{ds^{2}}$$

where n=0,1,2,…

By equating Equations (11) and (12), the energy eigenvalue is obtained.

## 3. Theory and Calculations

For spacetime with a point-like global monopole (PGM), the line element that explains it is given by [54,55]:

(13) $$ds^{2}=-c^{2}dt^{2}+\frac{dr^{2}}{\alpha^{2}}+r^{2}d\theta^{2}+r^{2}\sin^{2}\theta d\varphi^{2}$$

where $0<\alpha =1-8\pi G\eta_{0}^{2}<1$ is the parameter related to the PGM which depends on the energy scale $\eta_{0}$. Furthermore, the metric (13) portrays a spacetime with scalar curvature

(14) $$R=R_{\mu }^{\mu }=2\frac{\left(1-\alpha^{2}\right)}{r^{2}}$$

In this way, the Schrödinger equation (SE) is given as follows:

(15) $$-\frac{\hbar^{2}}{2\mu }\nabla_{LB}^{2}\psi \left(\vec{r},t\right)+V\left(r,t\right)\psi \left(\vec{r},t\right)=i\hbar \frac{\partial \psi \left(\vec{r},t\right)}{\partial t}$$

where μ is the particle’s mass, $\nabla_{LB}^{2}=\frac{1}{\sqrt{g}}\partial_{i}\left(\sqrt{g}g^{ij}\partial_{j}\right)$ with $g=\det\left(g_{ij}\right)$, is the Laplace–Beltrami operator and V(r,t)=V(r) is GMP (1). Thereby, the SE for the GMP in a medium with the presence of the PGM (1) is

(16) $$-\frac{\hbar^{2}}{2mr^{2}}\left[\alpha^{2}\frac{\partial }{\partial r}\left(r^{2}\frac{\partial }{\partial r}\right)+\frac{1}{\sin\theta }\frac{\partial }{\partial \theta }\left(\sin\theta \frac{\partial }{\partial \theta }\right)+\frac{1}{\sin^{2}\theta }\frac{\partial^{2}}{\partial \varphi^{2}}\right]\psi \left(r,\theta ,\varphi ,t\right)+V\psi \left(r,\theta ,\varphi ,t\right)=i\hbar \frac{\partial \psi \left(r,\theta ,\varphi ,t\right)}{\partial t}$$

In what follows, let us consider a particular solution to Equation (16) given in terms of the eigenvalues of the angular momentum operator ${\hat{L}}^{2}$ as

(17) $$\psi \left(r,\theta ,\varphi ,t\right)=e^{-i\frac{E_{n\ell }t}{\hbar }}\frac{U\left(r\right)}{r}Y_{\ell ,m}\left(\theta ,\varphi \right)$$

where $Y_{\ell ,m}\left(\theta ,\varphi \right)$ are spherical harmonics and R(r) is the radial wave function. Then, by substituting Equation (17) into Equation (16), we obtain radial wave equation

(18) $$\frac{d^{2}U\left(r\right)}{dr^{2}}+\left[\frac{2m}{\hbar^{2}\alpha^{2}}\left(E_{n\ell }-D_{0}{\left(1-e^{-\delta \left(r-r_{e}\right)}\right)}^{2}-D_{1}{\left(q-e^{-\delta \left(r-r_{e}\right)}\right)}^{2}\right)-\frac{\ell \left(\ell +1\right)}{\alpha^{2}r^{2}}\right]U\left(r\right)=0$$

Over the years, it has been known that equations of the form of (18) cannot be solved in the presence of the centrifugal term, $\frac{\ell \left(\ell +1\right)}{\alpha^{2}r^{2}}$. In a bid to conquer this hurdle, Pekeris [56] proposed an approximation scheme [56] to solve this problem. In view of this, to overcome the barrier in Equation (18), we adopt the Pekeris approximation scheme [56] to bypass with the centrifugal term:

(19) $$\frac{\hbar^{2}\ell \left(\ell +1\right)}{2\mu \alpha^{2}r^{2}}=\eta \left(C_{0}+C_{1}e^{-\beta x}+C_{2}e^{-2\beta x}\right)$$

where $x=\frac{r-r_{e}}{r_{e}}$ $\beta =\delta r_{e}$, $\eta =\frac{\hbar^{2}\ell \left(\ell +1\right)}{2\mu \alpha^{2}r_{e}^{2}}$ and $C_{i}$ is the parameter of coefficients i=0,1,2 and they are obtained as follows:

(20) $$\begin{array}{l} C_{0}=1-\frac{3}{\beta }+\frac{3}{\beta^{2}}\\ C_{1}=\frac{4}{\beta }-\frac{6}{\beta^{2}}\\ C_{2}=-\frac{1}{\beta }+\frac{3}{\beta^{2}} \end{array}$$

By using approximation in Equation (19) and using the change of coordinate $s=e^{-2\delta r}$, the radial part of the SE with the GMP reduces to

(21) $$\frac{d^{2}U\left(s\right)}{ds^{2}}+\frac{1}{s}\frac{dU\left(s\right)}{ds}+\frac{1}{s^{2}}\left[-\varepsilon_{n\ell }+\gamma_{0}s-\gamma_{1}s^{2}\right]U\left(s\right)=0$$

where

(22a) $$\begin{array}{l} -\varepsilon_{n\ell }=\frac{2\mu r_{e}^{2}}{\hbar^{2}\alpha^{2}\beta^{2}}\left(E_{n\ell }-D_{0}-D_{1}q^{2}-\eta C_{0}\right),\;\gamma_{0}=\frac{2\mu r_{e}^{2}}{\hbar^{2}\alpha^{2}\beta^{2}}\left(2D_{0}-2D_{1}q-\eta C_{1}\right)\\ \gamma_{1}=\frac{2\mu r_{e}^{2}}{\hbar^{2}\alpha^{2}\beta^{2}}\left(D_{0}+D_{1}+\eta C_{2}\right) \end{array}$$

Comparing (21) with the hypergeometric equation of Equation (2), we obtain the following polynomials:

(22b) $$\tilde{\tau }=1,\;\sigma \left(s\right)=s,\;\sigma^{2}\left(s\right)=s^{2},\;\tilde{\sigma }\left(s\right)=-\varepsilon_{n\ell }+\gamma_{0}s-\gamma_{1}s^{2}$$

The polynomial π(s) is given by,

(23) $$\pi \left(s\right)=\pm \sqrt{\gamma_{1}s^{2}+\left(k-\gamma_{0}\right)s+\varepsilon_{n\ell }}$$

To find the expression for k, the discriminant of (10) is equated to zero. Thus, we obtain,

(24) $$k=-\gamma_{0}\pm \sqrt{\gamma_{1}\varepsilon_{n\ell }}$$

The substituting k in π(s) in Equation (23),

(25) $$\pi \left(s\right)=\pm \left(\sqrt{\gamma_{1}}s-\sqrt{\varepsilon_{n\ell }}\right)$$

Taking the negative value of π(s) in Equation (25) to obtain,

(26) $$\pi^{\prime }\left(s\right)=-\sqrt{\gamma_{1}}$$

To obtain the polynomial τ(s), we use $\tau \left(s\right)=\tilde{\tau }\left(s\right)+2\pi \left(s\right)$

(27) $$\tau \left(s\right)=1-2\sqrt{\gamma_{1}}s+2\sqrt{\varepsilon_{n\ell }}$$

The derivative of τ(s) in Equation (27),

(28) $$\tau^{\prime }\left(s\right)=-2\sqrt{\gamma_{1}}<0$$

The parameter λ is defined as,

(29) $$\lambda =\gamma_{0}-2\sqrt{\gamma_{1}\varepsilon_{n\ell }}+\sqrt{\gamma_{1}}$$

$\lambda_{n}$ is expressed as,

(30) $$\lambda_{n}=2n\sqrt{\gamma_{1}}$$

The eigenvalue expression holds if

(31) $$\lambda =\lambda_{n}$$

(32) $$\varepsilon_{n\ell }={\left(\frac{\gamma_{0}}{2\sqrt{\gamma_{1}}}-\left(n+\frac{1}{2}\right)\right)}^{2}$$

Substituting Equation (22a) into (32) and evaluating it, we obtain the energy as follows:

(33) $$E_{n\ell }=D_{0}+D_{1}q^{2}+\frac{\hbar^{2}\ell \left(\ell +1\right)}{2\mu \alpha^{2}r_{e}^{2}}\left(1-\frac{3}{\beta }+\frac{3}{\beta^{2}}\right)-\frac{\hbar^{2}\beta^{2}\alpha^{2}}{2\mu r_{e}^{2}}{\left(\frac{\gamma_{0}}{2\sqrt{\gamma_{1}}}-\left(n+\frac{1}{2}\right)\right)}^{2}$$

To find the eigenfunction, the weight function is first evaluated. From Equation (7), we obtain

(34) $$\rho \left(s\right)=s^{2\sqrt{\varepsilon_{n\ell }}}e^{-2\sqrt{\gamma_{1}}s}$$

Integrating Equation (5), we obtain

(35) $$\phi \left(s\right)=s^{\sqrt{\varepsilon_{n\ell }}}e^{-\sqrt{\gamma_{1}}s}$$

Recalling y(s) is expressed in Rodrigues relation (5) and using (34), we obtain

(36) $$y_{{}_{n}}\left(s\right)=B_{n}\left(s\right)s^{-2\sqrt{\varepsilon_{n\ell }}}e^{2\sqrt{\gamma_{1}}s}\frac{d^{n}}{ds^{n}}\left[s^{n+2\sqrt{\varepsilon_{n\ell }}}e^{-2\sqrt{\gamma_{1}}s}\right]$$

The polynomial solution of $y_{n}$ in Equation (36) is expressed in terms of the associated Laguerre polynomials, which is one of the orthogonal polynomials, that is

(37) $$y_{{}_{n}}\left(s\right)=L_{n}^{2\sqrt{\varepsilon_{n\ell }}}\left(2\sqrt{\gamma_{1}}s\right)$$

Combining the Laguerre polynomials $y_{n}\left(s\right)$ and ϕ(s) in Equations (37) and (35), the radial wavefunction are constructed as

(38) $$\psi_{n\ell }\left(s\right)=B_{n\ell }s^{\sqrt{\varepsilon_{n\ell }}}e^{-\sqrt{\gamma_{1}}s}L_{n}^{2\sqrt{\varepsilon_{n\ell }}}\left(2\sqrt{\gamma_{1}}s\right)$$

## 4. Thermal Properties of Generalized Morse Potential (GMP)

All thermodynamic properties can be obtained from the system’s partition function, according to the extensive literature and basic text [33,34,35]. This means that a good evaluation of the system’s partition function would serve as the starting point for evaluating all of the system’s other thermal functions. An easy summation over all possible vibrational energy levels accessible to the system can be used to compute the vibrational partition function. Given the energy spectrum (33), the partition function Z(Λ) of the GMP at finite temperature T is obtained with the Boltzmann factor as [33,34,35]:

(39) $$Z\left(\Lambda \right)=\sum_{n=0}^{n_{\max}}e^{-\Lambda E_{n}}$$

with $\Lambda =\frac{1}{kT}$ and with k is the Boltzmann constant.

Substituting Equation (33) in (39), we have:

(40) $$Z\left(\Lambda \right)=\sum_{n=0}^{n_{\max}}e^{-\Lambda \left(D_{0}+D_{1}q^{2}+\frac{\hbar^{2}\ell \left(\ell +1\right)}{2\mu \alpha^{2}r_{e}^{2}}\left(1-\frac{3}{\beta }+\frac{3}{\beta^{2}}\right)-\frac{\hbar^{2}\beta^{2}\alpha^{2}}{2\mu r_{e}^{2}}{\left(\frac{\gamma_{0}}{2\sqrt{\gamma_{1}}}-\left(n+\frac{1}{2}\right)\right)}^{2}\right)}$$

where n is the vibrational quantum number, and $n=0,1,2,3\ldots n_{\max}$, $n_{\max}$ denotes the upper bound vibration quantum number. The maximum value $n_{\max}$ can be obtained by setting $\frac{dE_{n}}{dn}=0$. Converting the summation sign in (40) to an integral, it yields the following expression:

(41) $$Z\left(\Lambda \right)=\int_{0}^{n_{\max}}e^{-\Lambda \left(\xi -\chi {\left(\frac{\gamma_{0}}{2\sqrt{\gamma_{1}}}-\left(n+\frac{1}{2}\right)\right)}^{2}\right)}dn$$

where

(42) $$\begin{array}{l} \xi =D_{0}+D_{1}q^{2}+\frac{\hbar^{2}\ell \left(\ell +1\right)}{2\mu \alpha^{2}r_{e}^{2}}\left(1-\frac{3}{\beta }+\frac{3}{\beta^{2}}\right)\\ \chi =\frac{\hbar^{2}\beta^{2}\alpha^{2}}{2\mu r_{e}^{2}} \end{array}$$

If we set $t=\frac{\gamma_{0}}{2\sqrt{\gamma_{1}}}-\left(n+\frac{1}{2}\right)$, we can rewrite the above integral in Equation (34) as follows:

(43) $$Z\left(\Lambda \right)=-\int_{t_{1}}^{t_{2}}e^{-\Lambda \left(\xi -\chi t^{2}\right)}dt$$

where

(44) $$\begin{array}{l} t_{1}=t=\frac{\gamma_{0}}{2\sqrt{\gamma_{1}}}-\frac{1}{2},\\ t_{2}=\frac{\gamma_{0}}{2\sqrt{\gamma_{1}}}-\left(n_{\max}+\frac{1}{2}\right) \end{array}$$

Using Mathematica 9.0 to integrate the integral in Equation (43) yields the following vibrational partition function of the generalized Morse potential with topological defects:

(45) $$Z\left(\Lambda \right)=\frac{e^{-\Lambda \xi }\sqrt{\pi }\left(Erfi\left[t_{1}\sqrt{\Lambda }\sqrt{\chi }\right]-Erfi\left[t_{2}\sqrt{\Lambda }\sqrt{\chi }\right]\right)}{2\sqrt{\Lambda }\sqrt{\chi }}$$

where is Erfi is the error function. The classical partition function is represented by the above expression, Equation (45). All thermodynamic properties of the generalized Morse potential with topological defects can be obtained in the following, including the free energy, mean energy, entropy and specific heat capacity from the partition function. The following expressions can be used to calculate the thermodynamic functions of **LiH**, **TiC** and **I_2_** [33,34,35]:

(46) $$\begin{array}{l} F\left(\Lambda \right)=-\frac{1}{\Lambda }\ln Z\left(\Lambda \right),\\ U\left(\Lambda \right)=-\frac{d\ln Z\left(\Lambda \right)}{d\Lambda },\\ S\left(\Lambda \right)=\ln Z\left(\Lambda \right)-\Lambda \frac{d\ln Z\left(\Lambda \right)}{d\Lambda },\\ C\left(\Lambda \right)=\Lambda^{2}\frac{d^{2}\ln Z\left(\Lambda \right)}{d\Lambda^{2}}. \end{array}$$

Mathematica 9.0 is used to evaluate and plot the thermodynamic quantities.

## 5. Applications

In this section, the results obtained in the previous sections are used to study **LiH**, **TiC** and **I_2_** diatomic molecules in the presence of a topological defect. These diatomic molecules are selected because of their wide applications and studies by several authors. For instance, Oyewumi et al. [56] studied the thermal properties of these molecules with the shifted Deng–Fan potential. Ikot et al. [16] obtained the thermal properties of **LiH** using improved screened Kratzer potential. Again, Rampho et al. [57] studied the effects of external fields on the energy spectra, thermal and magnetic properties of **LiH** using improved screened Kratzer potential. We categorically state here that the authors of [57,58,59] studied **LiH**, **TiC** and **I_2_** diatomic molecules in the absence of a topological defect. However, we are inspired to scrutinize the effects of this defect on the energy spectra and thermal properties of these molecules. The experimental values of molecular constants for the lowest (i.e., ground) electronic state of the **LiH**, **TiC** and **I_2_** diatomic molecules are taken from the literature [56] and shown in Table 1 below. 

### 5.1. Energy Spectra

We used the following conversions: ℏc=1973.269 eVÅ and $1\;amu=931.5\times 10^{6}\mathrm{eV}{\left(Å\right)}^{-1}$ for all computations [58]. Table 2 shows the numerical energy spectra $E_{n\ell }\left(\mathrm{eV}\right)$ for **LiH**, **TiC** and **I_2_** molecules for different quantum states with various values of the topological and deformation parameter. It is observed that for a given quantum state, the energy decreases as the deformation parameter increases. However, if one pays close attention to the behavior of the energy spectra as it varies with the α, we immediately see that the energy increases with increasing α. This is consistent with the result of an earlier study by Marques and Bezerra [60]. 

**Figure 1:**
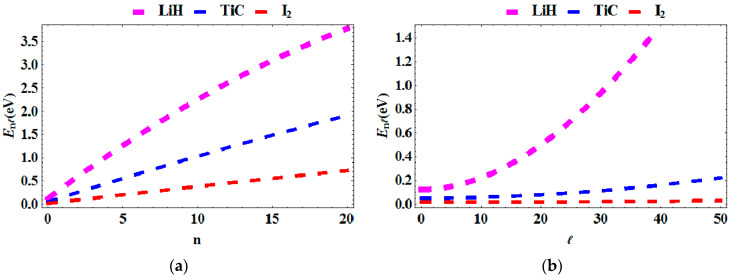

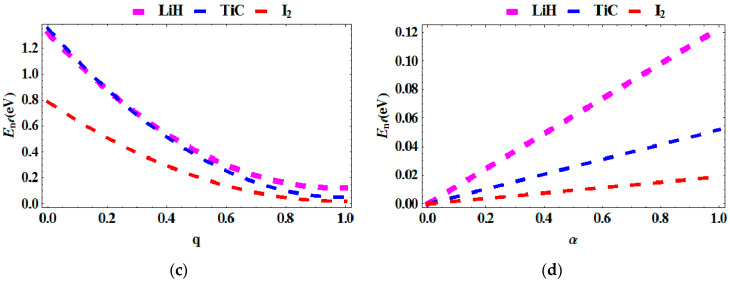
Energy spectra as a function of: (**a**) n for various diatomic molecules; (**b**) as a function of ℓ for various diatomic molecules; (**c**) as a function of q for various diatomic molecules; (**d**) as a function of α for various diatomic molecules.

In Figure 1a,b, the energy spectra is plotted as a function of the principal quantum number n, angular momentum ℓ, the deformation parameter q, and topological defect, α for various diatomic molecules. In Figure 1a, the energy is plotted against the principal quantum number, n. We see that, as the principal quantum number increases, the energy increases as well. However, upon comparison between the molecules, the molecule with highest energy is **LiH**, followed by **TiC** and then **I_2_**_._ In Figure 1b, the energy is plotted against the angular momentum, ℓ. The energy increases with increasing angular momentum for **LiH** but was quasi-constant for **TiC** and **I_2_** Figure 1c shows a plot of the energy versus the deformation parameter, q. The energy spectra are seen to decrease as the deformation parameter increases. This is observed in all three molecules studied. Figure 1d shows the energy of the system versus the topological defect. The energy of the system increases as the topological defect increases.

### 5.2. Partition Function

**Figure 2:**
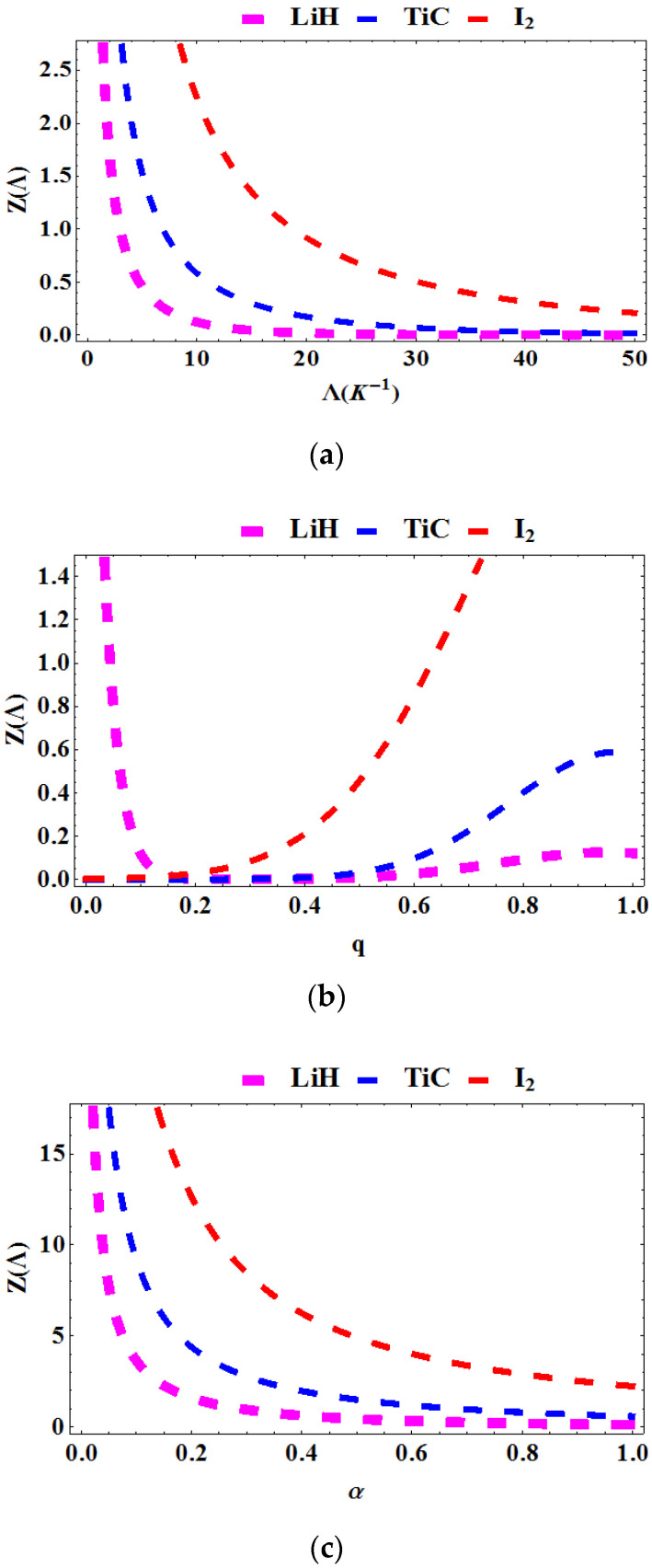
Partition function as a function of: (**a**) Λ for various diatomic molecules; (**b**) as a function of q for various diatomic molecules; (**c**) as a function of α for various diatomic molecules.

In Figure 2a–c, the partition function is plotted as a function of the Boltzmann factor $\Lambda =\frac{1}{kT}$, deformation parameter, q and topological defect, α for various diatomic molecules. In Figure 2a, the partition function is plotted against Λ. We see that as Λ increases, the partition decreases. In Figure 1b, the partition function is plotted against the deformation parameter, q. The partition function monotonically decreases as the q increases for the **LiH** diatomic molecule, but increases with rising temperature for **TiC** and **I_2_**. Figure 1c shows a plot of the energy versus the topological defect, α. The partition function is seen to decrease as the defect increases (i.e., α→1). This is observed in all three molecules studied. 

### 5.3. Free Energy

**Figure 3:**
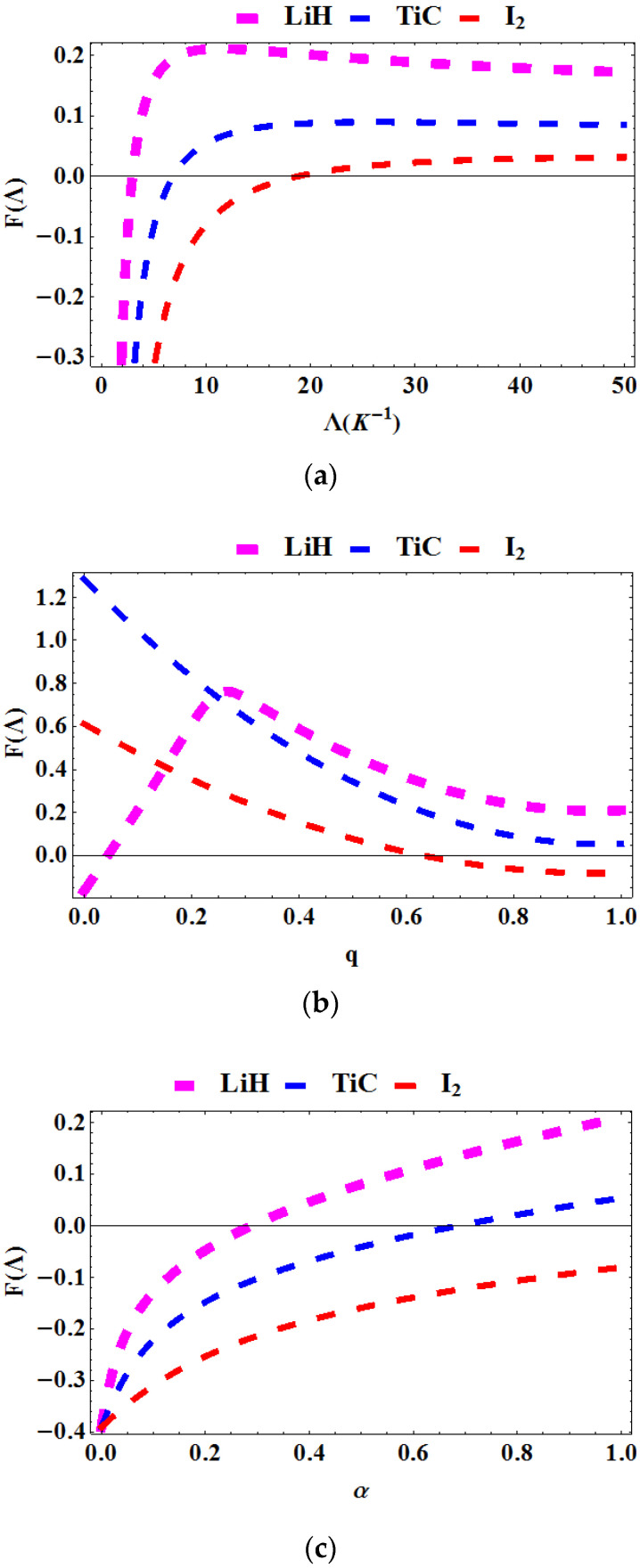
Free energy as a function of: (**a**) Λ for various diatomic molecules; (**b**) as a function of q for various diatomic molecules; (**c**) as a function of α for various diatomic molecules.

In Figure 3a–c, the free energy is plotted as a function of the Boltzmann factor $\Lambda =\frac{1}{kT}$, deformation parameter, q and topological defect, α for various diatomic molecules. In Figure 3a, the free energy is plotted against Λ. The free energy increases monotonically as the temperature increases. The free energy of **LiH** was found to be highest, followed by **TiC** and then **I_2_**. In Figure 3b, the free energy is plotted against q. The free energy for **LiH** first peaks in the interval 0<q<0.3 and then decreases rapidly beyond this region. However, for **TiC** and **I_2_**, the free energy decreases with increasing q for **I_2_**. In Figure 3c, the free energy is plotted against α. The free energy increases monotonically as the topological defect increases. 

### 5.4. Entropy

**Figure 4:**
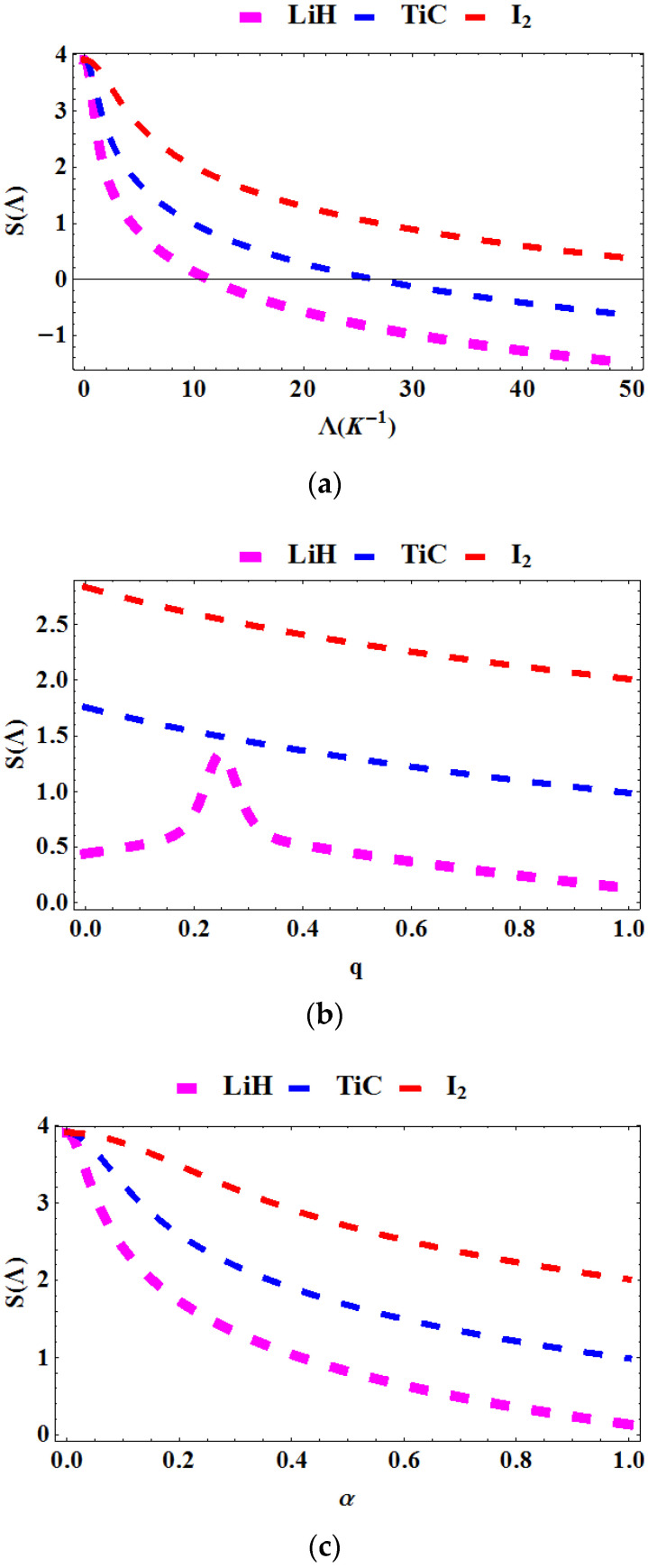
Entropy as a function of: (**a**) Λ for various diatomic molecules; (**b**) as a function of q for various diatomic molecules; (**c**) as a function of α for various diatomic molecules.

In Figure 4a–c, the entropy is plotted as a function of the Boltzmann factor $\Lambda =\frac{1}{kT}$, deformation parameter, q and topological defect, α for various diatomic molecules. In Figure 4a, the entropy is plotted against Λ. The entropy decreases with increasing Λ. In Figure 4b, the entropy is plotted against q. We see that the entropy decreases as the deformation parameter increases. Figure 4c shows a plot of the entropy against the topological defect. The entropy again decreases as the defect rises.

### 5.5. Average Energy

**Figure 5:**
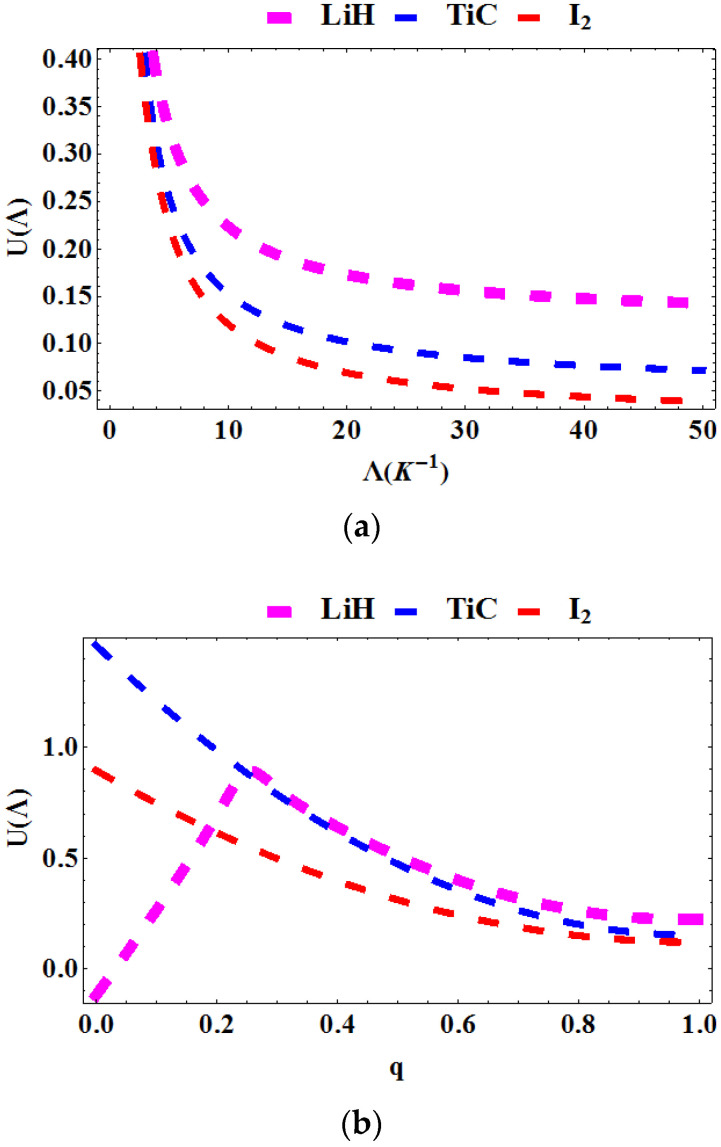

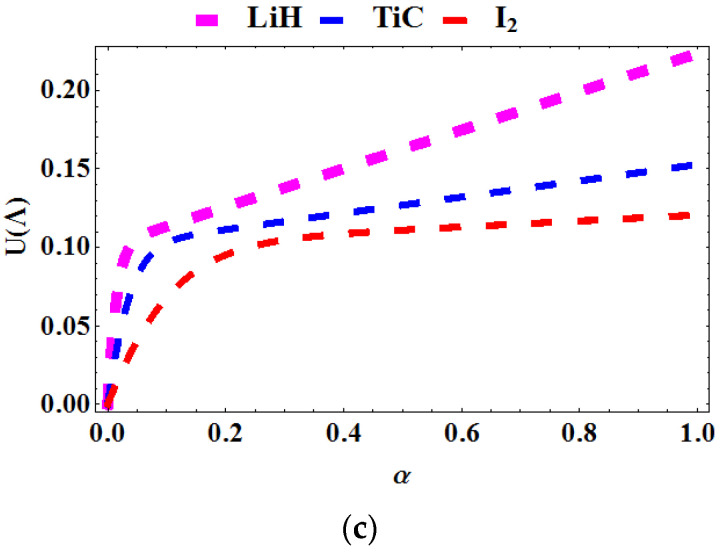
Average energy as a function of: (**a**) Λ for various diatomic molecules; (**b**) as a function of q for various diatomic molecules; (**c**) as a function of α for various diatomic molecules.

In Figure 5a–c, the average energy is plotted as a function of the Boltzmann factor $\Lambda =\frac{1}{kT}$, deformation parameter, q and topological defect, α for various diatomic molecules. In Figure 5a, the average energy is plotted against Λ. The average energy declines as the Λ increases. In Figure 5b, the average energy is plotted as a function of q for various diatomic molecules. The average energy for **LiH** first peaks in the interval 0<q<0.3 and then decreases rapidly beyond this region. However, for **TiC** and **I_2_**, the average energy decreases with increasing q. In Figure 5c, the average energy is plotted against α. The average energy increases monotonically as the topological defect increases. 

### 5.6. Specific Heat Capacity

**Figure 6:**
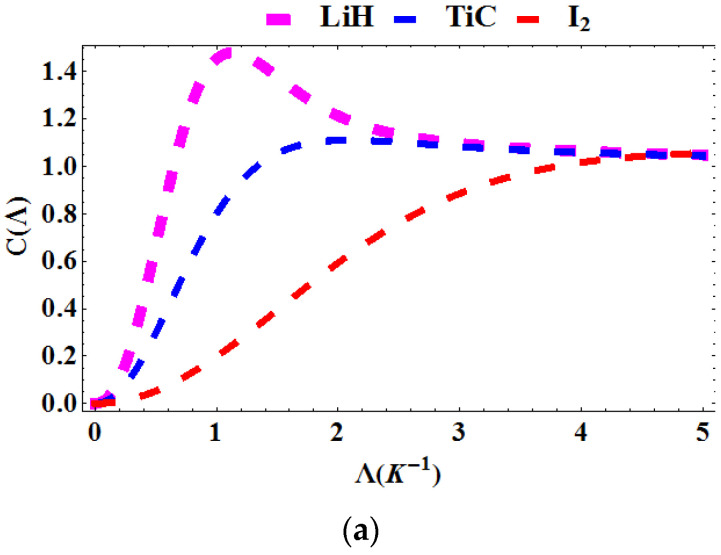

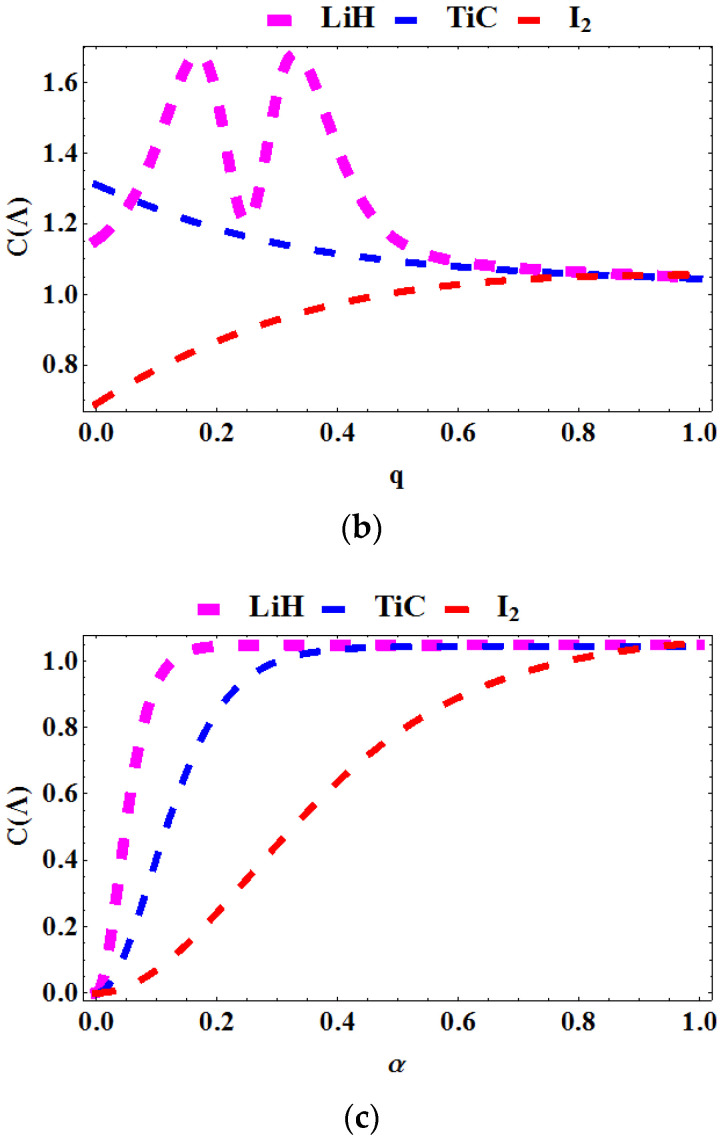
Specific heat capacity as a function of: (**a**) Λ for various diatomic molecules; (**b**) as a function of q for various diatomic molecules; (**c**) as a function of α for various diatomic molecules.

In Figure 6a–c, the specific heat capacity is plotted as a function of the Boltzmann factor $\Lambda =\frac{1}{kT}$, deformation parameter, q and topological defect, α for various diatomic molecules. In Figure 6a, the specific heat capacity is plotted against Λ. The specific heat capacity increases as Λ increases. The trend also reveals that the specific heat capacity saturates. In Figure 6b, the specific heat capacity is plotted as a function of q for various diatomic molecules. The specific heat capacity for **LiH** reveals a sinusoidal behavior as the deformation parameter varies. The specific heat capacity of **TiC** decreases with increasing q and that of **I_2_** increases with increasing q. In Figure 6c, the specific heat capacity is plotted against α. The specific heat capacity increases monotonically as the topological defect increases.

## 6. Conclusions

In this research article, we have scrutinized the effects of topological defect on the energy spectra and thermal properties of the generalized Morse potential for **LiH**, **TiC** and **I_2_** diatomic molecules. We see here that the presence of the defect and deformation can be used to alter the behavior of the system and its thermal properties. We also found that to create an upward shift in the energy spectra, the topological defect is required, whereas the deformation parameter can be used as a controller or an enhancer. The effects of the topological defect and deformation parameter on the thermal properties of the system is duly analyzed. We observe that the specific heat capacity of the system tends to exhibit quasi-saturation at large as the deformation parameter and topological defect approaches unity. Conclusively, we note here that our study of the generalized Morse potential in non-trivial gravitational background, such as what we treated in this study, will aid in the understanding of the problems of combining quantum mechanics and general relativity. The present model (GMP) could be applied for calculating the mass spectra of heavy mesons such as charmonium and bottomonium [61,62,63].

## Figures and Tables

**Table 1:** Spectroscopic constants of the diatomic molecules studied in the present work [56].

Molecules	$D_{0}=D_{1}=D_{e}$	$r_{e}\left(Å\right)$	$\delta \left({Å}^{-1}\right)$	μ(amu)
**LiH**	2.515267	1.5956	1.128	0.880122
**TiC**	2.66	1.79	1.5255	9.606079
**I_2_**	1.5556	2.662	1.8643	63.45224

**Table 2:** Energy spectra $E_{n\ell }\left(\mathrm{eV}\right)$ for **LiH**, **TiC** and **I_2_** molecules for different quantum states with various values of the topological and deformation parameter.

		LiH		TiC		I_2_	
State	α	q=0.5	q=1.0	q=0.5	q=1.0	q=0.5	q=1.0
1s	0.4	0.3512750	0.0491954	0.3480500	0.0207404	0.2001070	0.0075446
	0.8	0.3878990	0.0981492	0.3635600	0.0414403	0.2057560	0.0150801
	1.0	0.4061210	0.1225350	0.3713000	0.0517750	0.2085760	0.0188444
2s	0.4	0.4242820	0.1468610	0.3790290	0.0620996	0.2113950	0.0226064
	0.8	0.5319810	0.2915470	0.4251940	0.1238350	0.2282570	0.0451305
	1.0	0.5850140	0.3630740	0.4481390	0.1545660	0.2366570	0.0563616
2p	0.4	0.3599230	0.0608046	0.3487440	0.0215881	0.2001590	0.0076027
	0.8	0.3900530	0.1010420	0.3637330	0.0416519	0.2057680	0.0150946
	1.0	0.4074970	0.1243840	0.3714100	0.0519104	0.2085840	0.0188537
3s	0.4	0.4963230	0.2435600	0.4098460	0.1032970	0.2226450	0.0376316
	0.8	0.6721940	0.4810760	0.4861790	0.2055810	0.2506110	0.0750343
	1.0	0.7578630	0.5975680	0.5239660	0.2563430	0.2645080	0.0936497
3p	0.4	0.4328560	0.1583860	0.3797210	0.0629450	0.2114460	0.0226645
	0.8	0.5340970	0.2943980	0.4253660	0.1240450	0.2282700	0.0451449
	1.0	0.5863600	0.3648880	0.4482490	0.1547000	0.2366650	0.0563708
3d	0.4	0.3771840	0.0839731	0.3501330	0.0232833	0.2002630	0.0077189
	0.8	0.3943590	0.1068260	0.3640800	0.0420752	0.2057940	0.0151237
	1.0	0.4102470	0.1280790	0.3716320	0.0521811	0.2086010	0.0188723
4s	0.4	0.5673970	0.3392920	0.4405010	0.1443320	0.2338590	0.0526202
	0.8	0.8085400	0.6667380	0.5465170	0.2866790	0.2728190	0.1047920
	1.0	0.9246700	0.8260200	0.5987810	0.3571080	0.2921310	0.1307090
4p	0.4	0.5048230	0.2550000	0.4105360	0.1041400	0.2226970	0.0376896
	0.8	0.6742740	0.4838850	0.4863500	0.2057900	0.2506240	0.0750487
	1.0	0.7591800	0.5993490	0.5240750	0.2564770	0.2645170	0.0936590
4d	0.4	0.4499690	0.1813850	0.3811050	0.0646356	0.2115500	0.0227805
	0.8	0.5383290	0.3000960	0.4257100	0.1244660	0.2282950	0.0451739
	1.0	0.5890510	0.3685160	0.4484690	0.1549690	0.2366820	0.0563893
4f	0.4	0.4029900	0.1186020	0.3522150	0.0258259	0.2004190	0.0078931
	0.8	0.4008110	0.1154930	0.3645990	0.0427100	0.2058330	0.0151672
	1.0	0.4143700	0.1336200	0.3719640	0.0525871	0.2086260	0.0189001

**N/B**: α=1 means absence of topological defect.

## Data Availability

Data is contained within the article.

## List of Abbreviations

GMP	Generalized Morse potential
NU	Nikiforov–Uvarov
**LiH**	Lithium hydride
**TiC**	Titanium carbide
**I_2_**	Iodine
$\Lambda =\frac{1}{kT}$	Boltzmann factor
Z(Λ)	Partition function
F(Λ)	Free energy
S(Λ)	Entropy
U(Λ)	Average energy
C(Λ)	Specific heat capacity
T	Temperature
q	Deformation parameter
α	Topological defect (TD)
n	Vibrational quantum number
ℓ	Rotational quantum number
$E_{n\ell }\left(\mathrm{eV}\right)$	Energy spectra
